# Added Value of Three-Plane Multiecho Fast Field Echo MRI Sequence in the Evaluation of Acute Spinal Trauma Using Sensitivity: A Prospective Study

**DOI:** 10.7759/cureus.14694

**Published:** 2021-04-26

**Authors:** Deb K Boruah, Karuna Hazarika, Krishna K Borah, Halimuddin Ahmed, Barun K Sharma

**Affiliations:** 1 Radiodiagnosis, Tezpur Medical College, Tezpur, IND; 2 Radiodiagnosis, Sikkim Manipal Institute of Medical Science, Gangtok, IND

**Keywords:** magnetic resonance imaging (mri), emergency treatment, spinal cord, computed tomography (ct), gradient recalled echo (gre)

## Abstract

Background

Multiecho fast field echo (mFFE) MRI sequence provides added value to the conventional MR imaging in evaluation of acute spinal trauma, especially for detecting spinal cord hemorrhage which is a best predictor for patient prognosis.

Objective

This study aims to evaluate the diagnostic efficacies of three-plane mFFE MRI sequence along with the conventional MRI sequences in acute spinal trauma patients using sensitivity.

Materials and methods

This prospective study comprised of 48 patients of acute spinal trauma. The neurological deficit of acute spinal trauma patients assessed according to the American Spine Injury Association (ASIA) scale. The correlation between the various MRI findings of acute spinal cord injury and neurological deficits were compared with the Chi-square test.

Results

Of 48 patients of acute spinal trauma, 36 males and 12 females with a mean age of 38.71±1.42 [SD] years. 22 (45.8%) patients had cord edema with a mean length of was 3.45±5.52 [SD] cm. The mean percentage of spinal canal compromisation was 39.47±25.47 [SD] and spinal cord compression 18.1±24.4 [SD]. There was statistical significance between the ASIA impairment scale and spinal canal compromisation and cord compression with a p-value of 0.0005. Cord hemorrhage observed in 13 (27%), non-hemorrhagic cord contusions in 3 (6.3%), cord transection in 5 (10.4%) and epidural hematoma in 10 (20.8%) patients with an initial high grade of ASIA scale. The visibility score of three-plane mFFE sequence was higher in comparison to the single plane sagittal mFFE and short tau inversion recovery (STIR) sequences. For detection of spinal cord hemorrhage with visibility score of 2, the three-plane mFFE had sensitivity of 77% followed by 38.5% with single plane sagittal mFFE and 7.7% with sagittal STIR images. 26 (54.2%) patients showed neurological improvement in their hospital stay/follow-up period and no improvement observed in 7 (14.6%) patients of acute spinal trauma.

Conclusions

Application of three-plane mFFE sequences detects more spinal cord hemorrhages and vertebral fractures with a better visibility score as compared to the single sagittal plane mFFE and STIR sequence.

## Introduction

Plain radiograph and computed tomography scan are the initial imaging modalities of choice in acute spinal trauma. CT scan plays an important role in the rapid assessment of the acute spinal injury patient. However, MRI is the imaging modality of choice in acute spinal trauma patient which able to accurately detect the various vertebral fractures, ligamentous, disc, spinal cord and muscle injuries [1,2]. MRI precisely detect the various types of spinal cord injuries, which are missed on CT scan [1,2].

MRI plays a pivotal role in prognosticating acute spinal injury patients by detecting various pattern of acute spinal injuries, especially the spinal cord injuries [1,2]. Plain radiograph and CT scan fails to identify occult bone marrow, ligaments, muscle and spinal cord injuries, which can be readily detected by MRI. So, early detection of various acute spinal injuries with degree of severity of injuries impact early patient management and neurological outcome [3].

The MRI scan was usually subjected to a patient with acute spinal injury, when plain radiographic or CT findings are suspicious for ligamentous injury, to look for spinal cord injuries in patients with profound neurological deficit, to look for extra-medullary hemorrhage, post-traumatic disc herniation and spinal instability [2,4]. The acute spinal cord injuries vary from cord concussion, edema, contusion, hematoma to the transection [5]. 

MRI remains the mainstay of imaging modality in a suspected patient of acute spinal cord injury as MRI able to identify cord contusion, edema and hemorrhage [6-8]. Gradient recalled echo (GRE) sequence has a higher sensitivity to detect hemorrhage compared to a spin-echo sequence [6-8]. With the help of single or multi-plane GRE sequence like multiecho fast field echo (mFFE) sequence, MRI able to identify the degree and extent of cord hemorrhage and which helps the clinician to predict the patients functional and neurological recovery [6-10]. Previous literature showed poor neurological recovery, especially motor recovery in those patients, who had cord transection, hemorrhage and increased length of cord hemorrhage at initial MRI scan [9,11]. MRI is more sensitive in detecting occult bony injuries in vertebral body than posterior vertebral element because a lesser amount of cancellous bone in posterior vertebral element [12]. GRE sequence able to identify cord hemorrhage as dark signal intensity (blooming), extra-medullary hemorrhage and even vertebral body and posterior elements fractures [13]. The spinal cord hemorrhage appears as low signal intensity on T2WI with surrounding T2WI hyperintense edema. Three-plane mFFE sequence is the best for determining the location, size, extent of spinal cord hemorrhage which may be underestimated on conventional spin- echo MRI sequences [13].

The three-plane mFFE sequence can also detect ligament injury, muscle, paraspinal soft tissue injuries. mFFE can clearly differentiate hemorrhagic from non-hemorrhagic cord contusion [14-16]. Three-plane mFFE sequence able to demonstrate the displaced vertebral body or posterior element fractures, detect impingement over thecal sac, spinal cord and nerve root by retropulsed/displaced fractured bony fragment, detect facetal joint subluxation, intra- or extra-medullary hematoma at the level of injury [7,17]. Discontinuity of ligament can also be detected by mFFE sequence along with spin-echo T2W and short tau inversion recovery (STIR) sequences [18].

The prognostic markers of acute spinal injuries are degree of spinal canal compromisation and spinal cord compression, which can be measured by comparison at the site of injured segment, above and below the site of the injured segment [9].

This study aims to evaluate the diagnostic efficacies of three-plane mFFE MRI sequence along with the conventional MRI sequences in acute spinal trauma patients using sensitivity.

## Materials and methods

A prospective study was conducted at our institution on 48 patients of acute spinal trauma over a period of 12 months from August 2019 and July 2020 those underwent an MRI scan of the spine. This prospective study was approved by the Institutional ethics review committee. All acute spinal trauma patients underwent clinical examination during admission or before an MRI scan. The degree of neurological deficit of spinal trauma patients was determined according to the American Spine Injury Association (ASIA) impairment scale. The various MRI images were reviewed by two experienced radiologists blinded to the clinical information. Two radiologists observed for the vertebral body or posterior element fractures, cord edema, cord contusion, cord hemorrhage, cord transection or associated epidural hematoma. The neurological examinations were done by two experienced orthopedician. 

Correlation was done between the various MRI findings of spinal injuries and neurological deficit.

Patient selection

**Figure 1:**
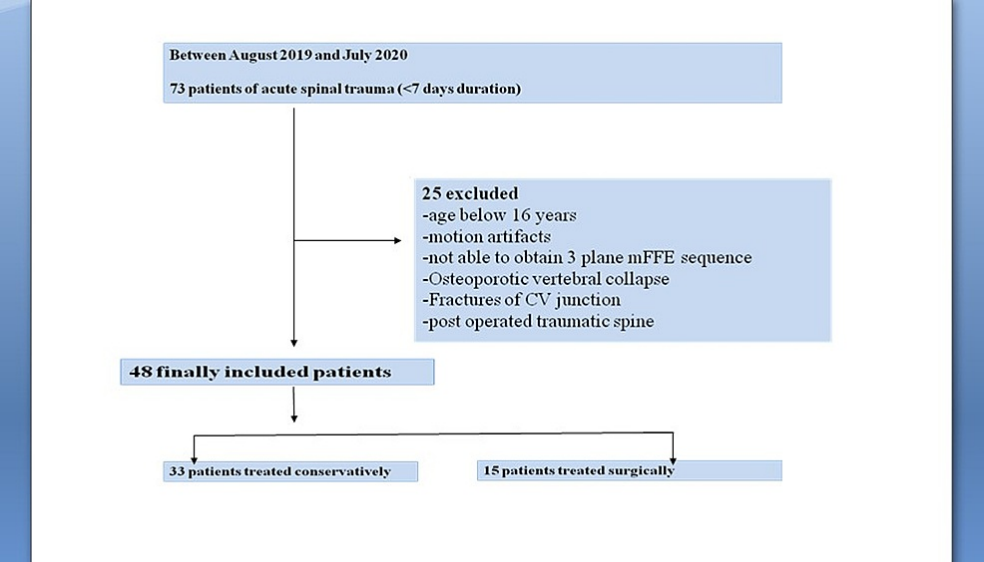
Flow diagram of the study population.

Acute spinal trauma patients with a history of injury less than 7 days of duration were included and finally 48 patients, male = 36 (75%) and female = 12(25%) with a mean age of 38.71±1.42 [SD] years were included in the study (Figure 1).

Exclusion criteria

1. Spinal trauma more than seven days of duration.

2. Children age below 16 years of age.

3. Pathological vertebral fracture.

4. Osteoporotic vertebral collapse.

5. Post operated traumatic spine.

6. Cerebral vertebral junction fractures

MRI protocols

All patients underwent an MRI scan of the spine, using a 1.5 T MR scanner, Philips Ingenia (Philips Medical System, the Netherlands). The MRI sequences protocol includes sagittal T2WI, T1WI, STIR, DWI, coronal STIR, axial T1WI, T2WI followed by three-plane mFFE sequences. The parameters of the various MRI sequences are shown in Table 1.

**Table 1:** Parameters used in various MRI sequences for spinal injury patients. STIR: short tau inversion recovery; mFFE: multiecho fast field echo; TE: time of echo; TR: repetition time; TI: inversion time.

MRI sequence	TE (ms)	TR (ms)	Matrix	Field of view (FOV)	Slice thickness (mm)	Flip angle	Others
T1W sagittal	10-12	500-600	220 x 280	200-220	4	90^0^	
T2W sagittal	90-110	2800-4200	220 x 280	200-220	4	90^0^	
STIR sagittal	60-90	2900-4300	220 x 280	200-220	4	90^0^	TI=150 ms
STIR coronal	60-90	2600-4100	220 x 280	200-220	4	90^0^	TI=150 ms
DWI sagittal	15-20	3500-4400		200-220	4	90^0^	b=1,000 sec/mm^2^
T1W axial	8-12	500-600	150 x 90	200-220	4	90^0^	
T2W axial	90-110	2800-4200	150 x 90	200-220	4	90^0^	
mFFE axial, coronal and sagittal	8-9	700-800	220x 152	200-220	3	25^0^-28^0^	2 min 31 sec in each plane

MRI evaluation

MRI images were evaluated for the site, level, pattern of the vertebral fractures, status of intervening disc, ligaments, ligamentum flavum , para-spinal muscles and various spinal cord injuries (SCI) like cord edema, cord contusion, cord hemorrhage and cord transection. 

The maximum spinal canal and cord compromise were calculated in the mid-sagittal T1WI images using these formulae:

Spinal canal compromise (%) = (1- ­­­­­­­­­­­­­­­­­­­­­­­Di/(Da+Db)/2) x 100.

Di = diameter of spinal canal at the injured site, Da = diameter above the injured segment and Db= diameter below the injured vertebral segment.

Spinal cord compression (%)=( 1- ­­­­­­­­­­­­­­­­­­­­­­­di/(da+db)/2) x 100.

di = diameter of cord at the injured site, da = diameter above the injured segment and db = diameter below the injured segment.

Degree of maximum spinal cord compromise measured in vertebral fractures of C2 to L1 vertebral level. In patient with multiple vertebral fractures, the maximum site of spinal canal and cord compromisation was selected for the measurement.

Visibility score of cord hemorrhage

The visibility of the spinal cord hemorrhage was categorised on a 3-point scale from 0 to 2. Score 0 = no cord hemorrhage detected, 1 = probably present cord hemorrhage and 2 = distinctly present cord hemorrhage. To know the diagnostic performance of 3 plane mFFE sequence in the evaluation of spinal cord hemorrhage, the imaging findings were determined on single plane (sagittal) mFFE images and three-plane mFFE images .

Neurological assessment of acute spinal trauma

The degree of neurological deficit of spinal trauma patients were determined according to the American Spine Injury Association (ASIA) impairment scale. Scale A (complete)- No sensory or motor function is preserved in the sacral segments S4-5, Scale B(Sensory incomplete)- Sensory but not motor function is preserved below the neurological level and includes sacral segments S4-5, Scale C (Motor incomplete)- Motor function is preserved below the neurologic level, and more than half of the key muscles below the neurologic level have a muscle grade <3, Scale D(Motor incomplete)-Motor function is preserved below the neurologic level, and at least half of key muscles below the neurologic level have a muscle grade of 3 or more and Scale E(Normal)- Sensory and motor functions are normal.

Statistical analysis

All statistical analysis was performed using Statistical Package for the Social Science (SPSS, version 16) (IBM Corp., Armonk, NY). Chi-square test was used to find out the correlation between the various spinal cord injuries on MRI with the neurological deficits. One-way ANOVA test was used find out the correlation between the maximum spinal canal and cord compromisation with the neurological deficits.

## Results

Patients demography

The study group comprised of 48 patients, male = 36 (75%) and female = 12 (25%) with a mean age of 38.71±1.42 [SD] years. Road traffic accident (RTA) was the most common cause of acute spinal injury in 29 (60.4%) patients followed by fall from height in 14 (29.2%) patients and slipped injury in 5 (10.4%) patients. The mean duration from the day of injury to the day of MRI scan was 4.19±2.3 [SD] days. Cervical vertebral fractures observed in 8 (16.7%) patients followed by dorsal vertebra in 15(31.3%) patients and lumbar vertebra in 18 (37.5%) patients. Multi-level vertebral fractures observed in 6 (12.5%) patients. Solitary vertebral fracture was observed in 28 (58.3%) patients, two vertebrae in 10 (20.8%) patients, three vertebrae in two patients (4.2%) and more than three vertebrae involvement in 5 (10.4%) patients.

ASIA impairment scale

Seven (14.6%) patients had ASIA scale A followed by scale B in 9 (18.8%) patients, scale C in 8 (16.7%) patients, scale D in 9 (18.8%) patients and scale E in 15 (31.2%) patients at the time of hospital admission or before an MRI scan.

Vertebral findings

**Figure 2:**
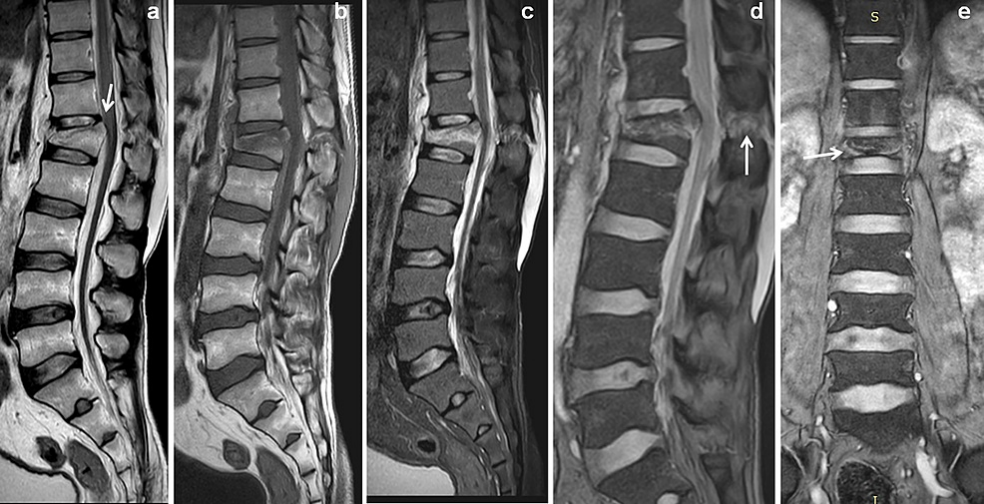
Vertebral compression collapse. 42-year-old male patient with history of fall from height. Sagittal T2 W and T1W images (a & b) showed compression collapse with fractures of L1 vertebra with retropulsion into spinal canal (↓arrow). Sagittal STIR and mFFE images (c & d) showed tearing of ALL, buckling of PLL and tearing of inter and supraspinous ligaments (↑arrow). Coronal mFFE image (e) showed the hyperintense fracture lines in the collapsed the vertebra (→ arrow). STIR: short tau inversion recovery; mFFE: multiecho fast field echo.

**Figure 3:**
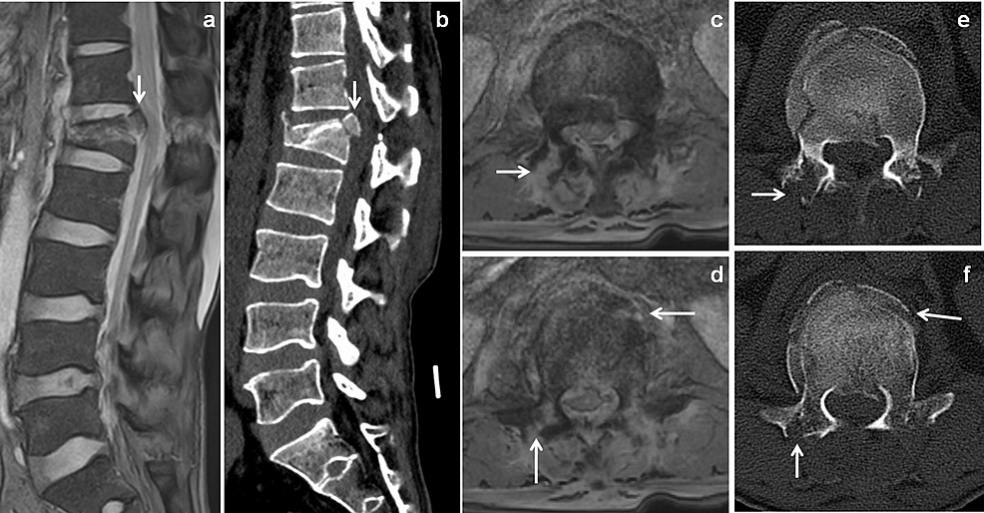
Vertebral compression collapse with CT scan correlation. Same patient of Figure 2 comparing with mFFE images with CT scan. Sagittal mFFE and sagittal reconstructed CT scan images (a & b) showed the L1 vertebral fractures with retropulsion of postero-superior margin into spinal canal (↓arrow). Axial mFFE images (c & d) and axial CT scan images (e & f) showed displaced fractures of vertebral body (←arrow) and posterior vertebral elements (↑ arrow). STIR: short tau inversion recovery; mFFE: multiecho fast field echo.

**Figure 4:**
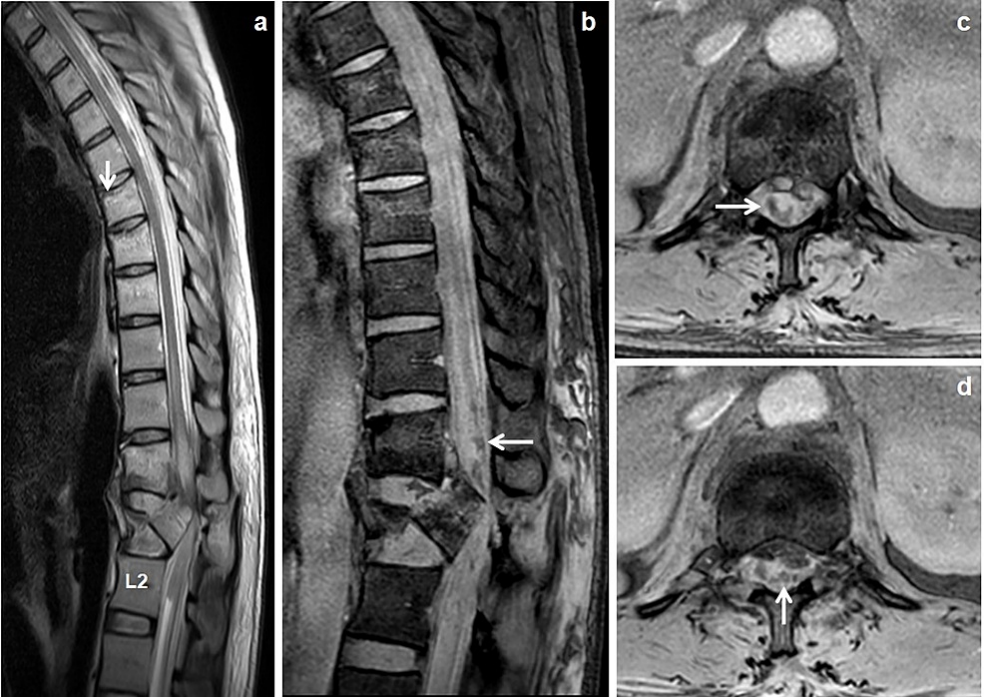
Vertebral burst fractures. Day 1 MRI scan of 41-year-old female patient with history of RTA. Sagittal T2W image (a) showed displaced burst fractures of L1 vertebra retropulsed into spinal canal and teared the longitudinal ligaments, crushed injury to the adjacent intervertebral discs and partial cord transection with long segmental cord edema up to D10 vertebral level. Occult fractures/bone contusions observed in D7, D8 and D9 vertebrae (↓arrow). Sagittal mFFE image (b) showed extensive cord hemorrhages in the lower dorsal cord (←arrow). Axial mFFE images (c & d) showed hemorrhages within the central (↑arrow) and para-central (→) locations of the cord. mFFE: multiecho fast field echo; RTA: road traffic accident.

Conventional MRI sequences along with three plane mFFE sequences identified various fractures of vertebral body and posterior elements. Out of 48 patients, 46 (95.8%) patients had vertebral fractures. Various types of vertebral body and posterior elements fractures were shown in (Table 2). Compression fractures of the vertebral body were most commonly observed in 24(50%) patients (Figures 2, 3) followed by burst fractures in 16 (33.3%) patients (Figure 4). There was statistical significance between the ASIA impairment scale and types of vertebral body fractures with a p-value of 0.019.

**Table 2:** MRI findings of acute spinal trauma in 48 patients at the time of admission/before MRI scan according to ASIA impairment scale. ASIA: American Spine Injury Association; RTA: road traffic accident.

MRI findings			ASIA impairment scale					Total	p-value
			A	B	C	D	E		
Mean age (years)			34.57±10.39 [SD]	30.44±8.06 [SD]	34.12±11.67 [SD]	37.89±11.57 [SD]	48.53±16.79 [SD]	38.71±1.42 [SD]	0.014
Sex	Male		6	8	7	8	7	36 (75%)	0.053
	Female		1	1	1	1	8	12 (25%)	
Mean duration (day) to the day of MRI scan			2.57±1.72 [SD]	3.11±2.36 [SD]	3.25± 2.25 [SD]	5.11±2.32 [SD]	5.53±1.6 [SD]	4.19± 2.3 [SD]	0.0005
Mode of injury	RTA		6	5	4	5	9	29 (60.4%)	0.023
	Fall from height		1	4	4	4	1	14 (29.2%)	
	Slipped injury		0	0	0	0	5	5 (10.4%)	
Site of vertebral fracture	Cervical		2	3	3	0	0	8 (16.7%)	0.0005
	Dorsal		2	2	0	0	11	15 (31.3%)	
	Lumbar		0	2	4	8	4	18 (37.5%)	
	Mixed		3	1	1	1	0	6 (12.5%)	
Vertebral body fracture/injury	Compression fracture		3	3	0	5	13	24 (50%)	0.019
	Burst fracture		3	3	5	4	1	16 (33.3%)	
	Occult fracture/bone contusion		1	1	0	0	1	3 (6.3%)	
	Avulsion fracture		0	0	2	0	0	2 (4.2%)	
	No fracture		0	1	1	0	0	2 (4.2%)	
Posterior element fractures	Spinous process		1	1	1	0	0	5 (10.4%)	0.514
	Articular facets	Unilateral	1	0	0	0	0	1 (2.1%)	0.001
		Bilateral	5	2	2	0	0	9 (18.8%)	
	Facetal joint	Subluxation	1	0	1	0	0	2 (4.2%)	0.002
		Dislocation	5	2	1	0	0	8 (16.7%)	
	Transverse process	Unilateral	4	2	0	1	0	7 (14.6%)	0.013
		Bilateral	0	0	1	0	0	1 (2.1%)	
	Lamina	Unilateral	3	1	1	0	1	6 (12.5%)	0.089
		Bilateral	2	2	1	1	0	6 (12.5%)	
	Pedicle	Unilateral	1	2	0	2	0	5 (10.4%)	0.193
		Bilateral	2	2	2	2	0	8 (16.7%)	
Supraspinous ligament injury	Sprain		0	1	0	1	0	2 (4.2%)	0.066
	Partial tear		1	0	0	1	0	2 (4.2%)	
	Complete tear		5	2	3	2	1	13 (27.1%)	
Interspinous ligament injury	Sprain		1	2	2	3	2	10 (20.8%)	0.047
	Partial tear		1	0	0	1	0	2 (4.2%)	
	Complete tear		5	2	3	1	1	12 (25%)	
ALL tear	Buckled		1	2	2	3	7	15 (31.3%)	0.196
	Partial Tear		0	1	1	3	3	8 (16.7%)	
	Complete tear		6	3	4	3	3	19 (39.6%)	
PLL tear	Buckled		2	3	4	7	8	24 (50%)	0.038
	Partial tear		0	0	0	0	1	1 (2.1%)	
	Complete tear		5	3	1	1	0	10 (20.8%)	
Ligamentum flavum tear	Partial tear		2	0	2	1	0	5 (10.4%)	0.006
	Complete tear		5	4	2	2	1	14 (29.2%)	
Intervertebral disc injury	Contusion		1	2	2	4	9	18 (37.5%)	0.119
	Crushed		1	2	2	4	4	13 (27.1%)	
	herniation		5	3	3	1	1	13 (27.1%)	
Paraspinal muscle injury	Muscle sprain		4	4	5	4	1	18 (37.5%)	0.008
	Muscle tear		2	1	0	0	0	3 (6.3%)	

Spinal cord abnormality

**Figure 5:**
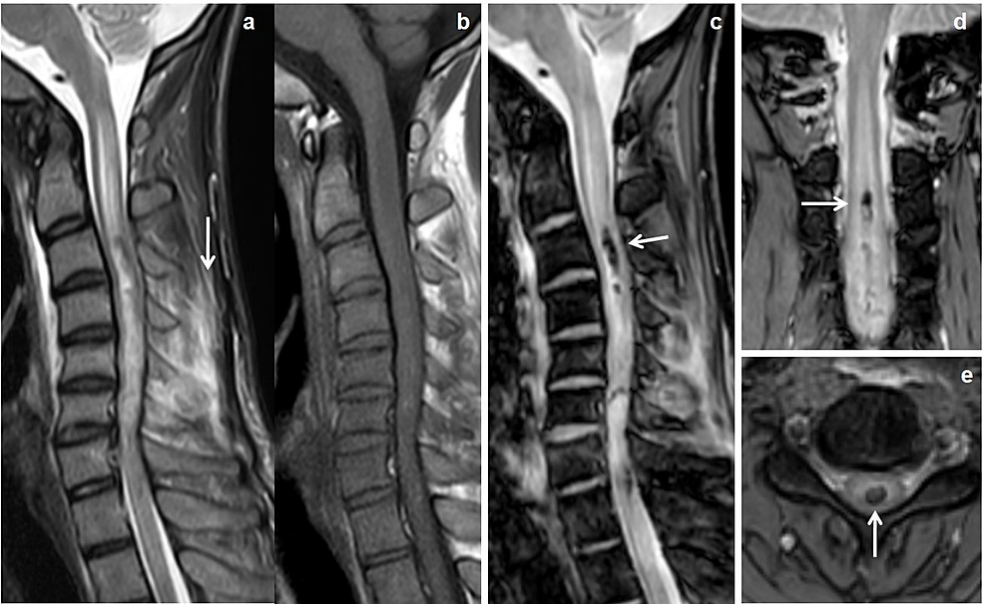
Cervical cord hemorrhage. Day 2 MRI scan of a 30-year-old male patient with history of RTA had quadriplegia. Sagittal STIR and T1W images (a & b) showed T2 hyperintense long segment cervical cord edema extends from cervico-medullary junction to C7 vertebral level with STIR hyperintensities in lower cervical inter and supraspinous ligaments (↓arrow). Sagittal mFFE image (c) showed multi-level cervical cord hemorrhages (← arrow). Coronal and axial mFFE images (d & e) showed central cervical cord hemorrhages (→ & ↑ arrows). STIR: short tau inversion recovery; mFFE: multiecho fast field echo; RTA: road traffic accident.

**Figure 6:**
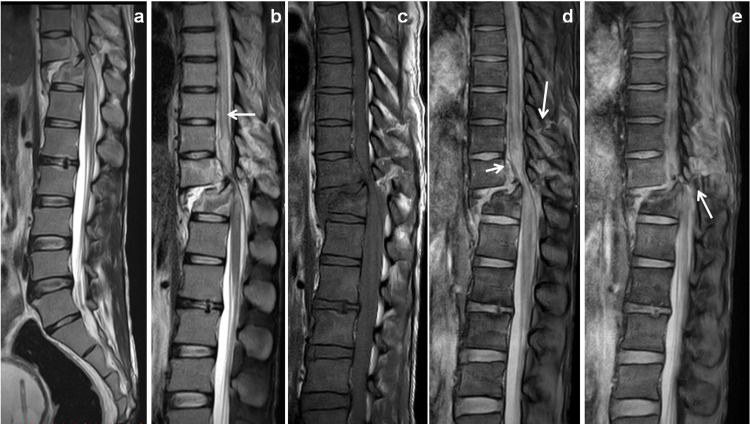
Displaced vertebral fracture with near-total cord dissection. Day 2 MRI scan of 28-year-old male patient with history of RTA. Sagittal T2W, STIR and T1W images (a, b & c) showed displaced fractures of D12 vertebra with teared longitudinal ligaments, ligamentum flavum and long segmental dorsal cord edema extends up to D7 vertebral level (← arrow). Sagittal mFFE images (d & e) showed ventral epidural hematoma (→ arrow). Hyperintense fractures noted in the D9, D10, D11 and D12 vertebrae (↓ arrow). Near-total cord transection was noted with teared posterior ligamentous complex affecting ligamentum flavum, Interspinous and supraspinous ligaments (↑ arrow). STIR: short tau inversion recovery; mFFE: multiecho fast field echo; RTA: road traffic accident.

Out of 48 patients, spinal cord abnormalities identified in 22 (45.8%) patients. Various spinal cord abnormalities were shown in (Table 3). Spinal cord edema observed in 22 (45.8%) patients with or without cord hemorrhage (Figure 5). The mean length of cord edema was 3.45±5.52 [SD] cm. There was statistical significance between the ASIA impairment scale and length of cord edema with a p-value of 0.003. Only spinal cord edema observed in 6 (12.5%) patients, where three in the cervical cord, two in the conus medullaris and one in the thoraco-lumbar cord. Cord hemorrhage was observed in 13 (27%) patients, where four in the cervical cord (Figure 5), four in the dorsal cord, two in conus medullaris, two in cervico-thoracic cord and one in the thoraco-lumbar cord. Non-hemorrhagic cord contusion was observed in 3 (6.3%) patients, where one in cervical cord, one in thoraco-lumbar cord and another one in conus medullaris. Cord transection was observed in 5 (10.4%) patients, where three had partial cord transection ( Figure 4), one had near-total transection (Figure 6) and another one had complete cord transection.

**Table 3:** Salient MRI findings and neurological status according to ASIA impairment scale in 48 patients of acute spinal trauma at the time of admission/ before MRI scan. ASIA: American Spine Injury Association.

MRI Findings	ASIA impairment scale					Total	p-value
	A	B	C	D	E		
Number of patient	7 (14.6%)	9 (18.8%)	8 (16.7%)	9 (18.8%)	15 (31.2%)	48	
Normal cord	0	0	2	9	15	26 (54.2%)	0.0005
Isolated cord edema	1	3	2	0	0	6 (12.5%)	
Cord haemorrhage	6	6	1	0	0	13 (27%)	
Non-haemorrhagic cord contusion	0	0	3	0	0	3 (6.3%)	
Mean length of cord edema (cm)	13.2±3.4 [SD]	5.56±6.02 [SD]	2.9±3.3 [SD]	0	0	3.45±5.52 [SD]	0.003
Cord transection	5	0	0	0	0	5 (10.4%)	0.001
Mean of maximum spinal canal compromisation (%)	67.1±9.9 [SD]	31.3±28.3 [SD]	38.59±26.69 [SD]	55.92±19.4 [SD]	22.11±13.57 [SD]	39.47±25.47 [SD]	0.0005
Mean of maximum spinal cord compression (%)	65±14.18 [SD]	24.1±18.75 [SD]	15.12±16.68 [SD]	0	4.94±7.2 [SD]	18.1±24.4 [SD]	0.0005
Epidural haematoma	5	3	2	0	0	10 (20.8%)	0.001

Sagittal mFFE, STIR and three-plane mFFE visibility score of cord hemorrhage

Thirteen out of 48 patients of acute spinal trauma had spinal cord hemorrhage on mFFE sequence shown in (Table 4). Out of 13 patients, cord hemorrhage was distinctly visualized (visibility score 2) in 10 (76.9%) patients on three-plane mFFE sequences (Figure 5) and 5 (38.5%) patients on single plane sagittal mFFE sequence. Detection of spinal cord hemorrhage with visibility score of 2, the three-plane mFFE had a sensitivity of 77% followed by 38.5% with single plane sagittal mFFE and 7.7% with sagittal STIR images.

**Table 4:** Visibility score of spinal cord hemorrhage of sagittal mFFE, STIR and three-plane mFFE sequences in 13 out of 48 patients of acute spinal trauma. STIR: short tau inversion recovery; mFFE: multiecho fast field echo; ASIA: American Spine Injury Association.

MRI sequence	Visibility score	Cord haemorrhage (n = 13)	ASIA impairment scale			p-value (chi-square)
			A	B	C	
Sagittal mFFE	Score 2	5	3	1	1	0.194
	Score 1	5	3	2	0	
	Score 0	3	0	3	0	
Three-plane mFFE	Score 2	10	6	3	1	0.103
	Score 1	3	0	3	0	
	Score 0	0	0	0	0	
Sagittal STIR	Score 2	1	1	0	0	0.749
	Score 1	9	4	4	1	
	Score 0	3	1	2	0	

Out of 13 patients, cord hemorrhage was poorly visualized (visibility score 1) in 3 (23%) patients on three-plane mFFE sequences and 5 (38.5%) patients on single plane sagittal mFFE sequence. Detection of spinal cord hemorrhage with visibility score of 1 or above , the three-plane mFFE had sensitivity of 100% followed by 77% with single plane sagittal mFFE and 61.5% with sagittal STIR images.

No significant correlation found between the visibility scores of cord hemorrhage and ASIA impairment scale shown in (Table 4).

Maximum spinal canal compromisation and cord compression

In 48 patients, the mean percentage of spinal canal compromisation was 39.47±25.47 [SD] and spinal cord compression 18.1±24.4 [SD] from the fractured vertebra, epidural hematoma or both. The mean percentage of maximum spinal canal compromisation was 67.1±9.9 [SD] in ASIA scale A patients followed by 55.92±19.4[SD] in ASIA scale D patients. Mean of maximum percentage of spinal cord compression was 65±14.18 [SD] in ASIA scale A followed by 24.1±18.75[SD] in ASIA scale B patients . There was statistical significance between the ASIA impairment scale and spinal canal compromisation and cord compression with a p-value of 0.0005.

Ligaments and soft tissue injuries

Various ligaments and soft tissue injuries shown in Table 3. Injuries to supraspinous ligament were identified in 17(35.4%) patients, interspinous ligament in 24 (50%) patients, anterior longitudinal ligament in 27 (56.3%) patients (Figure 3), posterior longitudinal ligament in 11 (22.9%) patients (Figure 4) and ligamentum flavum in 19 (39.6%) patients.

Management

Out of 48 patients of acute spinal trauma, 33 patients treated conservatively and 15 patients treated surgically.

Neurological recovery

The neurological recovery after treatment shown in (Table 5). In our study sample, seven patients had initial ASIA scale A, only 2 (28.6%) showed neurological improvement, one patient to ASIA scale B and another one patient to ASIA scale C. Rest of five patients of ASIA scale A did not show neurological improvement. three patients of ASIA scale B improved to ASIA scale C, four patients to ASIA scale D while two patients did not show any neurological improvement. six patients of ASIA scale C improved to ASIA scale D and two patients to ASIA scale E. All nine patients of ASIA scale D improved to scale E.

**Table 5:** Patients neurological recovery after the treatment according to the ASIA impairment scale. ASIA: American Spine Injury Association.

ASIA impairment scale at admission/before MRI scan	Conservative	Surgical treatment	Improvement	Non-improvement	Not applicable	Total number of cases
A	1	6	2	5		7
B	5	4	7	2		9
C	4	4	8	0		8
D	8	1	9	0		9
E	15	0	-	-	15	15

In this study sample of 48 patients, 26 (54.2%) patients showed improvement in neurological status, seven patients (14.6%) showed no improvement and 15 (31.3%) patients had no neurological deficit at the time of admission/before MRI scan.

## Discussion

Three-plane mFFE sequence was highly accurate in diagnosing spinal cord hemorrhage which helps in prognosticating acute spinal cord injury (SCI) in comparison to single plane mFFE sequence [14,15]. Application of three-plane mFFE sequences can better identify the cord hemorrhage, various vertebral body and posterior element fractures which may in some situations obviate the need for a CT scan [13]. In our study sample of 48 patients, the majority of patients being males (75%). Young- and middle-aged men were the most common age group involved with a mean age of 38.71±1.42[SD] years. Similar trend reported by Singh et al. [3].

RTA was the comment cause of acute spinal injuries in 29 (60.4%) patients followed by fall from height in 14 (29.2%) and slipped injury in 5 (10.4%) patients. Singh et al. [3] reported that a fall from height was more common.

Parashari et al. [16] and Magu et al. [19] commonly found acute spinal trauma patients of ASIA scale A while in our study sample ASIA scale E was found in 15 (31.2%) patients and least in ASIA scale A in 7(14.6%) patients. This was probably because of more number of patients of thoraco-lumbar injuries in our study sample. 

mFFE sequence can also able to detect extra-medullary hemorrhage. Kerslake et al. [18] found epidural hematoma in 41% of patients of acute spinal trauma with a higher incidence in ankylosed spine [20]. In our study sample, 10 (20.8%) patients had an epidural hematoma.

Injuries of cervical spine were commonly observed by Fehlings et al. [21] followed by lower dorsal and upper lumbar spinal injuries. Cervical spine injury is a common site because of excessive mobility during RTA or fall from height and lack of supporting structures observed by Gupta et al. [22]. Looby et al. [23] reported more thoraco-lumbar injuries than cervical spine injuries. In our study sample, most commonly thoraco-lumbar injuries observed in 25 (52%) patients followed by cervical vertebral injuries in 10 (20.8%) patients.

The degree of spinal canal compromisation and cord compression act as an important factor for spinal cord injury [9] and which also act as a prognostic indicator for neurological deficit [23]. In our study, the mean of maximum percentage of spinal canal compromisation was 67.1±9.9 [SD] in ASIA scale A followed by 55.92±19.4 [SD] in ASIA scale D and spinal cord compression was 65±14.18 [SD] in ASIA scale A followed by 24.1±18.75 [SD] in ASIA scale B.

Kulkarni et al. [7] described three patterns of MRI appearances that prognosticate acute spinal cord injury are cord hemorrhage, cord edema, combination of cord hemorrhage and edema. In our study sample, the visibility score of three-plane mFFE sequences was higher in comparison to the single plane sagittal mFFE sequence for detecting cord hemorrhage shown in (Table 4).

Bondurant et al. [24] described four patterns of spinal cord injuries. Normal cord in Pattern 1, cord edema for a single vertebral level in Pattern 2, multiple vertebral level cord edema in Pattern 3 and mixed cord hemorrhage and edema in Pattern 4. Changes in signal abnormalities of cord edema found within the first two weeks following acute spinal cord injury [25].

In our study sample, spinal cord abnormalities found in 22 (45.8%) patients. All 22 patients showed cord edema with a mean length of 3.45±5.52[SD]cm followed by cord hemorrhage in 13 (27%) patients and cord transection in 5(10.4%) patients. Post-traumatic spinal cord edema was also commonly observed by Parashari et al. [16]. Parashari et al. [16] observed cord abnormalities in 75.8% of patients and Kulkarni et al. [7] in 70.4% of patients.

In our study sample of 48 patients, 26 (54.2%) patients showed improvement in their neurological status, 7 (14.6%) patients showed no improvement and the remaining 15 (31.3%) patients had no neurological deficit at the time admission/ before an MRI scan. No neurological improvement observed in four patients of cord transection and another four patients of cord hemorrhage throughout for hospital stay/follow-up. Previous literatures observed good neurological recovery in patients with non-hemorrhagic cord contusion or cord edema as compared to cord hemorrhage [8,24,26]. No neurological improvement was observed in patients with cord hemorrhage by Gupta et al. [22] and cord transection by Qiu et al. [27]. In our study, 11 patients with normal cord findings on MRI scan with a neurological deficit of ASIA scale C and scale D showed complete neurological recovery. Patients with initial ASIA scale A have lower chances of recovery (28.6%), whereas maximum chances of recovery were associated with ASIA scale D (100%). Harrop et al. [28] observed 7% neurological improvement in patients with initial ASIA scale A and 94.3% improvement in ASIA scale D.

Out study observed a more detection with higher visibility score of spinal cord hemorrhage, extra-medullary hematoma and vertebral body and posterior element fractures with institution of three-plane mFFE sequences as compared to the single plane sagittal mFFE and STIR sequences. However, no statistically significant difference was observed between these. Therefore, a larger prospective study to confirm these findings is warranted in the future. Another limitation of three-plane mFFE sequence was more time-consuming in comparison to a single plane mFFE sequence of 2 min 31 sec.

## Conclusions

MRI is the choice of investigation for the patient with acute spinal injury. MRI with institution of three-plane mFFE sequence can define the spinal cord hemorrhage, vertebral fractures and facetal articulations abnormalities. MRI appearance of length of cord edema, cord hemorrhage, and spinal cord transection are the important considerations in neurological deficit and which helps in planning early patient management.
